# A meta-analysis on RCTs of direct anterior and conventional approaches in total hip arthroplasty

**DOI:** 10.1038/s41598-021-00405-4

**Published:** 2021-10-25

**Authors:** Nikolai Ramadanov, Simon Bueschges, Philip Lazaru, Dobromir Dimitrov

**Affiliations:** 1 Department of Emergency Medicine, University Hospital Jena, Friedrich Schiller University, Am Klinikum 1, 07747 Jena, Germany; 2 Faculty of Medicine, Department of Statistics, University of Salamanca, Calle Espejo 2, 37007 Salamanca, Spain; 3 Center for Surgery, Evangelical Hospital Ludwigsfelde-Teltow, Albert-Schweitzer-Str. 40-44, 14974 Ludwigsfelde, Germany; 4 Department of Surgical Propaedeutics, Faculty of Medicine, Medical University of Pleven, Bulgaria, ul. Sveti Kliment Ohridski 1, 5800 Pleven, Bulgaria

**Keywords:** Rehabilitation, Fracture repair

## Abstract

To conduct a systematic review and meta-analyses on short-term outcomes between total hip arthroplasty (THA) through direct anterior approach (DAA) compared to THA through conventional approaches (CAs) in treatment of hip diseases and fractures. We performed a systematic literature search up to March 1, 2021 to identify RCTs, comparing THA through DAA with THA through CAs. We calculated mean differences (MDs) with 95% confidence intervals (CIs) for continuous outcomes, using the DerSimonian and Laird method and a random effects model. We calculated odds ratios (ORs) with 95% CIs for dichotomous outcomes, using the Mantel–Haenszel method and a random effects model. Ten RCTs met the criteria for final meta-analysis, involving 1053 patients. Four studies were blinded RCTs with a level I evidence, the other 6 studies were non-blinded RCTs with a level II evidence. DAA had a longer operation time than CAs (MD = 17.8, 95% CI  4.8 to 30.8); DAA had similar results compared to CAs for incision length (MD = − 1.1, 95% CI − 4.1 to 1.8), for intraoperative blood loss (MD = 67.2, 95% CI − 34.8 to 169.1), for HHS 3 months postoperatively (MD = 2.4, 95% CI − 0.7 to 5.5), for HHS 6 months postoperatively (MD = 0.8, 95% CI − 1.9 to 3.5), for HHS 12 months postoperatively (MD = 0.9, 95% CI − 0.7 to 2.5), for pain VAS 1 day postoperatively (MD = − 0.9, 95% CI − 2.0 to 0.15), for acetabular cup anteversion angle (MD = − 4.3, 95% CI − 5.2 to − 3.5), for acetabular cup inclination angle (MD = − 0.5, 95% CI − 2.1 to 1.1) and for postoperative complications (OR = 2.4, 95% CI 0.5 to 12.4). Considering the overall results of our meta-analysis, we can conclude that THA through DAA showed similar short-term surgical, functional, radiological outcomes and postoperative complications compared to THA through CAs.

## Introduction

Several systematic reviews and meta-analyses on short-term outcomes between total hip arthroplasty (THA) through direct anterior approach (DAA) compared to THA through conventional approaches (CAs)^1–9^ came to contradicting conclusions. While some of them found an advantage of DAA^2–4,6–8^, others showed indifferent results^1,5,9^. Most of those meta-analyses had relevant limitations. The 2015 meta-analysis by Higgins et al.^1^, comparing DAA with posterior approach, included 17 studies (2 RCTs, 10 retrospective, and 5 non-randomized prospective studies) with a total of 2302 patients. The 2019 meta-analysis by Jia et al.^2^, comparing DAA with posterior approach, included 20 studies (4 RCTs, 3 prospective studies and 13 retrospective studies) with a total of 7377 patients. The 2015 meta-analysis by Yue et al.^7^, the first meta-analysis comparing DAA with lateral approach, included 12 studies (2 RCTs and 10 non-RCTs) with 2,991 cases of THA through DAA and 1,910 cases of THA through lateral approach. Unfortunately, the inclusion of studies with a low level of evidence in these meta-analyses^1,2,7^ affects the validity of the end point estimates and overall results. The 2019 meta-analysis by Kucukdurmaz et al.^3^, comparing DAA with other approaches, included 18 RCTs with a total of 1,661 patients. Only this meta-analysis considered the influence of traction (fracture) table utilization in THA through DAA^3^. Unfortunately, navigated THA and THA through mini-incision approaches were pooled in one group, which presents a severe confounding factor. Furthermore, three relevant recent RCTs^10–12^ were not included in final meta-analysis. The 2018 meta-analysis by Miller et al.^4^, comparing DAA with posterior approach, included 13 prospective studies (7 RCTs and 6 non-RCTs) with 1,044 patients. This meta-analysis had a very short follow-up period of 3 months. Furthermore, some outcome parameters were grouped together, but measured at very different time points. The 2018 meta-analysis by Wang et al.^6^, the first meta-analysis comparing DAA with posterior approach, included 9 RCTs with a total of 854 patients. It was performed with high quality methods. The 2018 meta-analysis by Putananon et al.^5^, the first network meta-analysis comparing outcomes of different surgical approaches in THA, included 14 RCTs with a total of 1,201 patients. It investigated the outcomes of THA through DAA, lateral, posterior and posterior-2-incision approaches. This network meta-analysis limited their investigation to three outcome parameters: VAS, HHS and postoperative complications. Posterior and posterior-2-incision approaches were pooled based on only two studies. The systematic reviews by Kyriakopoulos et al. and Meermans et al. did not perform a meta-analysis^8,9^. In order to overcome these limitations of the related previous systematic reviews and meta-analyses^1–9^, a new meta-analysis was required with a more extensive literature search, with restriction to RCTs and with implementation of high-quality statistical methods.

Our aim was to compare short-term outcomes of THA through DAA and THA through CAs in treatment of hip diseases and femoral neck fractures by performing a systematic literature review and meta-analysis of RCTs.

## Methods

### Literature search and study selection

Our review protocol was registered and approved with PROSPERO on 28 February 2021 (CRD42021233481). We followed the PRISMA-P guidelines to report our meta-analysis. Our search was performed up to March 1, 2021 in the following databases: PubMed, Embase, Web of Science, The Cochrane Library, SCOPUS, CINAHL, Clinical Trials. We used a BOOLEAN search strategy [(DAA) OR (direct anterior approach)], adapted to the syntax of the used databases. Furthermore, we checked citations of screened studies and Google Scholar for additional records. Titles and abstracts were screened to identify articles for further consideration, full text of the selected articles were screened again according to inclusion criteria. The study selection was determined by the consensus of two independent reviewers (NR and PL). Kappa coefficient was used to measure the agreement between the reviewers.

### Inclusion/exclusion criteria and outcomes

Inclusion criteria were as follows: RCTs with no restriction to language and publication date, comparing outcomes in THA through DAA and THA through CAs. We included human participants with the following hip pathologies: osteoarthritis, avascular necrosis of the femoral head, dysplasia, femoral neck fracture. We excluded studies on THA through mini-incision approaches as well as surgical techniques, using a computer navigation system. The types of measured outcomes were the following: The operation time in min. was defined as the period of time from the beginning of skin incision to surgical closure. The incision length in cm was measured on graduated scale. The intraoperative blood loss in ml was the total amount of blood from the suction device. The pain Visual Analogue Scale (VAS)^13,14^ ranges from 0 to 10 points, with the pain intensity increasing with the number of points. Hip pain was evaluated at day 1 after surgery. The Harris Hip Score (HHS)^15^ ranges from 0 to 100 points, with hip function increasing with the number of points. It was evaluated at periodic time intervals after surgery. The acetabular cup anteversion and the inclination have ideal values for positioning: anteversion angle from 10° to 25° and inclination angle from 35° to 45°.

### Data extraction and study quality assessment

We extracted data on study characteristics, methods, quality assessment, characteristics of participants, details of the interventions, and measured outcomes into a standard electronic spreadsheet. We contacted corresponding authors for missing data. In case that relevant data was still missing, the concerning study was excluded in order to guarantee a high quality inclusion of RCTs. Risk of bias and level of evidence assessment were performed according to the Cochrane’s Risk of Bias 2 (RoB 2) tool^16^ and the guidelines of the Centre for Evidence-Based Medicine^17^.

### Statistical analysis

DAA represented the “experimental group” and CAs represented the “control group”. We tested both fixed and random effects models. The random effects model provided more reliable results, so we proceeded as follows: We calculated mean differences (MDs) with 95% confidence intervals (CIs) for continuous outcomes, using the DerSimonian and Laird method and a random effects model. We calculated odds ratios (ORs) with 95% CIs for dichotomous outcomes, using the Mantel–Haenszel method and a random effects model. A common τ^2^ was assumed for calculation of the random effects estimates. Study weighting was performed by inverse variance^18^. We evaluated the results and analysed them on basis of the Cochrane Handbook for Systematic Reviews of Interventions^19^. We did not pool study data that were clinically too diverse. Heterogeneity was assessed using a test on Cochrane’s Q statistic, which followed a distribution with k-degrees of freedom (p value < 0.10 is indicative of heterogeneity), and a Higgins’ test I^2^ (low heterogeneity, < 25%; moderate heterogeneity, 25–75%; and high heterogeneity, > 75%)^20^. We performed a traction table subgroup-analysis in order to examine the influence of traction table utilization in THA through DAA.

## Results

### Study identification and selection

**Figure 1:**
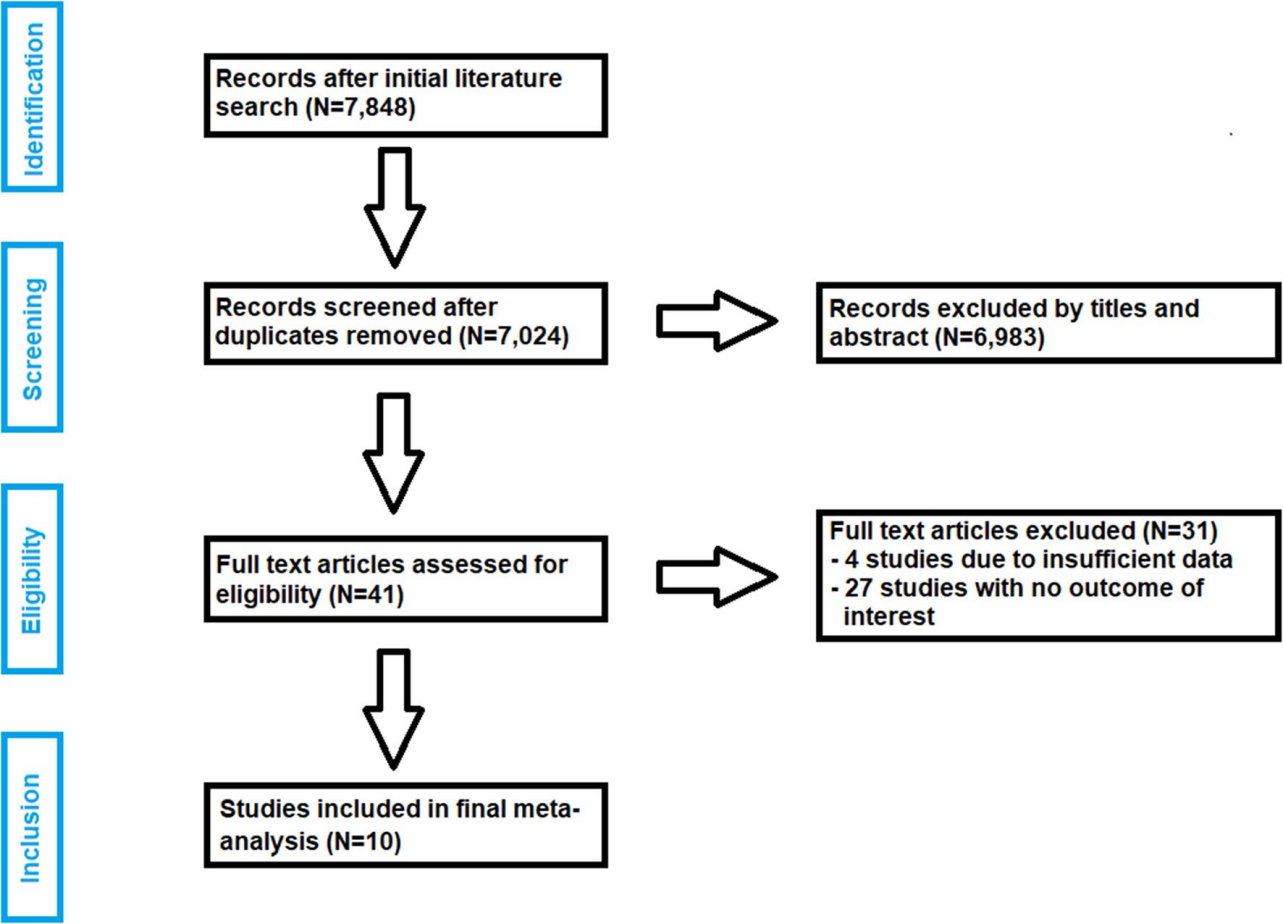
PRISMA flow diagram of the search results and selection according to our inclusion criteria. *DAA* direct anterior approach, *RCTs* randomized controlled trials.

After removal of 824 duplicates, a total of 7,024 studies were found in initial literature search. Forty-one studies were assessed for eligibility after first screening procedure by title and abstract with disagreement between the reviewers concerning one study (κ = 0.98). Thirty-one studies were excluded after full-text screening according to inclusion criteria (κ = 1.0). Four of those studies were excluded because they did not provide any information on standard deviation of the outcome parameters examined^21–24^. A total of 10 studies on THA through DAA met the criteria for final meta-analysis^10–12,25–31^. Details of study identification, screening, and selection are given in a PRISMA flow diagram (Fig. 1).

### Characteristics of the RCTs

Table 1 gives an overview of the main characteristics of the 10 included RCTs, published between 2009 and 2020, altogether involving 1,053 patients with 1,057 operated hip joints. Of these, 468 were operated through DAA and 585 through CAs. The sample size of the 10 studies ranged from 46 to 169 patients, all published in English language. Of the 10 studies, 4 included conventional THA through posterolateral approach^10,25,30,31^, one through posterior approach^12^, 5 through lateral transgluteal approach^11,26–29^. Three studies reported to have used traction table in THA through DAA^10,12,25^. Only one study included patients with bilateral THA^31^.

**Table 1:** Main characteristics of RCTs included in network meta-analysis.

Study	Sample size, n		Surgical approach		Mean age, y (SD or range)		Gender (M/F), n		BMI, kg/m^2^ (SD or range)		Hip pathology				Follow-up period, months	Origin of study
	Pts	Hips	DAA	CAs	DAA	CAs	DAA	CAs	DAA	CAs	Osteoarthritis	ANFH	Dysplasia	Femoral neck fracture		
Barrett et al. 2013	87	87	43TT	44 pl	61,4 ± 9,2	53,2 ± 7,7	29/14	19/25	30,7 ± 5,4	29,1 ± 5	87	–	–	–	12	USA
Bon et al. 2019	100	100	50 TT	50 pl	67,26 ± 10	68,98 ± 7,93	21/29	23/27	26,46 ± 3,58	26,69 ± 3,12	100	–	–	–	3	France
D'Arrigo et al. 2009	169	169	20	149 l	64 ± 8	65 ± 9,8	12/8	81/68	22,7 ± 1,5	28 ± 1,8	169	–	–	–	1,5	Italy
De Anta-Diaz et al. 2016	99	99	49	50 l	63,5 ± 12,5	64,8 ± 10,1	26/23	26/24	26,9 ± 3,1	26,6 ± 3,9	99	–	–	–	12	Spain
Mjaaland et al. 2015	163	163	83	80 l	67,2 ± 8,6	65,6 ± 8,6	25/58	30/50	3,6 ± 1,9	27,6 ± 3,9	163	–	–	–	< 1	Norway
Moerenhout et al. 2020	55	55	28 TT	27 p	70,4 ± 9,1	68,9 ± 8,8	11/28	18/27	26,5 ± 4,3	27,6 ± 4,4	55		–	–	55	Canada
Nistor et al. 2017	70	70	35	35 l	67	64	26/9	16/19	27,45 ± 3,76	38,63 ± 3,12	70	–	–	–	3	Romania
Reichert et al. 2018	148	148	77	71 l	63,2 ± 8,2	61,9 ± 7,8	45/32	71/0	28,1 ± 3,7	28,3 ± 3,4	148	–	–	–	12	Germany
Rykov et al. 2017	46	46	23	23 pl	62,8 ± 6,1	60,2 ± 8,1	8/15	11/12	29 ± 5,6	29,3 ± 4,8	46	–	–	–	1,5	Netherlands
Zhao et al. 2017	116	120	60	56 pl	64,9 ± 12,1	62,2 ± 14,7	24/36	22/34	24,35 ± 3,1	25,58 ± 2,83	81	26	13	–	6	China

*DAA* direct anterior approach, *TT* traction table, *CAs* conventional approaches, *pl* posterolateral approach, *p* posterior approach, *l* lateral approach, *Pts* patients, *ANFH* aseptic necrosis of the femoral head.

### Risk of bias and level of evidence

Four out of 10 studies were rated with a low risk of bias^10,12,28,31^, 4 studies with a moderate risk of bias^11,26,27,29^ and 2 studies with a high risk of bias^25,30^ as shown in Table 2. Four out of 10 studies were blinded RCTs with a level I evidence^10,12,28,31^, the other 6 studies were non-blinded RCTs with a level II evidence^11,25–27,29,30^.

**Table 2:** Risk of bias assessment.

Study	Random sequence generation	Allocation concealment	Blinding	Complete outcome data	No selective reporting	No other sources of bias	Overall risk of bias
Barrett et al. 2013	Y	N	N	U	Y	U	High RB
Bon et al. 2019	Y	Y	Y	Y	Y	Y	Low RB
D'Arrigo et al. 2009	Y	Y	U	Y	Y	U	Moderate RB
De Anta-Diaz et al. 2016	Y	Y	U	Y	Y	Y	Moderate RB
Mjaaland et al. 2015	Y	Y	Y	Y	Y	Y	Low RB
Moerenhout et al. 2020	Y	Y	Y	Y	Y	Y	Low RB
Nistor et al. 2017	Y	Y	U	Y	Y	U	Moderate RB
Reichert et al. 2018	Y	Y	U	Y	Y	U	Moderate RB
Rykov et al. 2017	Y	Y	N	Y	Y	U	High RB
Zhao et al. 2017	Y	Y	Y	Y	Y	Y	Low RB

*DAA* direct anterior approach, *Y* Yes, *U* Unclear, *N* No, *RB* risk of bias.

### Outcomes

#### Operation time

**Figure 2:**
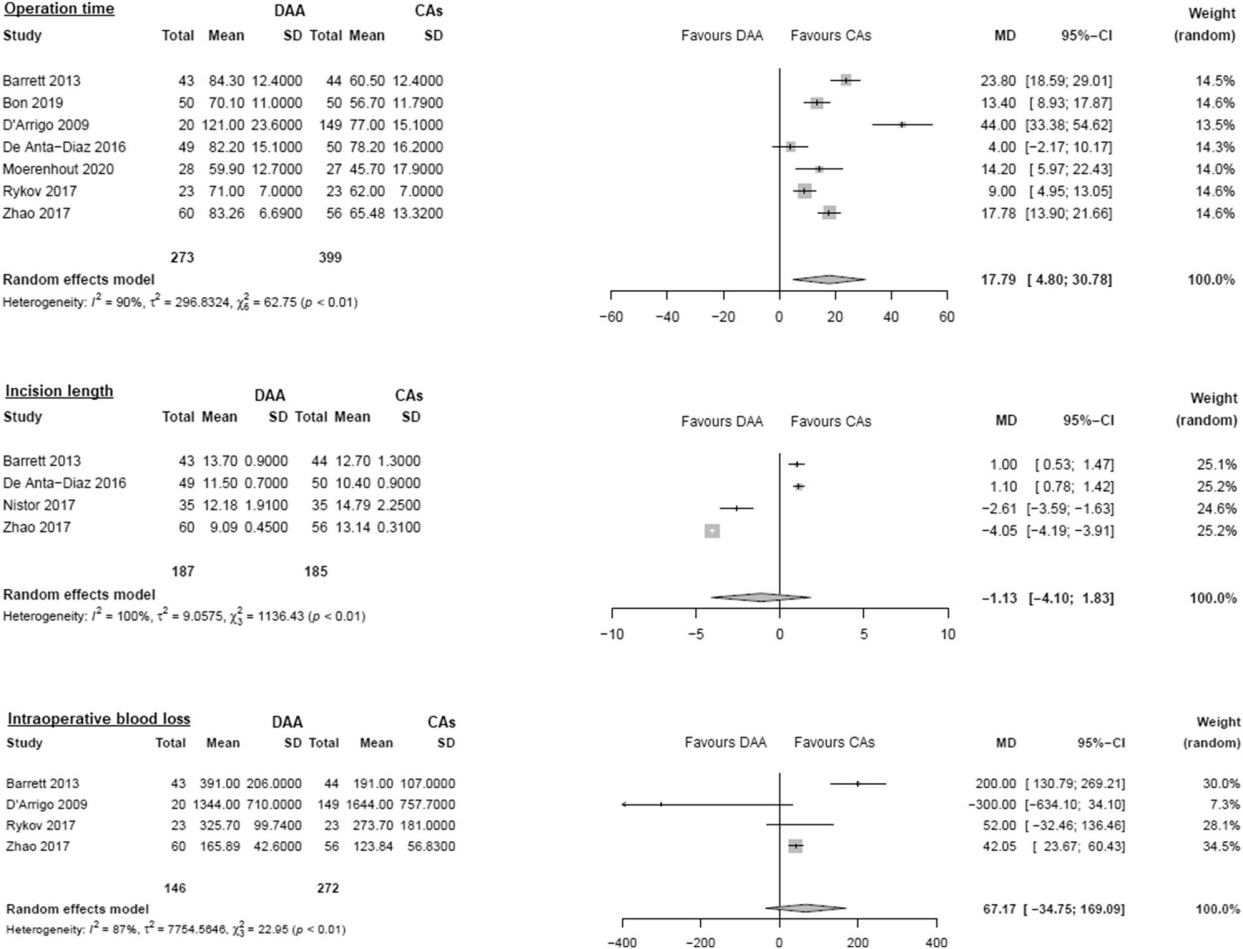
Comparison of the operation time, incision length, intraoperative blood loss. *DAA* direct anterior approach, *CAs* conventional approaches, *SD* standard deviation, *MD* mean difference, *CI* confidence interval.

Data on 672 patients were pooled from 7 RCTs (I^2^ = 90%, p < 0.01, Fig. 2). The operation time of THA through DAA was 17.8 min. longer than the operation time of THA through CAs (MD = 17.8, 95% CI 4.8 to 30.8).

#### Incision length

Data on 372 patients were pooled from 4 RCTs (I^2^ = 100%, p < 0.01, Fig. 2). There was no difference in incision length (MD = − 1.1, 95% CI − 4.1 to 1.8).

#### Intraoperative blood loss

Data on 418 patients were pooled from 4 RCTs (I^2^ = 87%, p < 0.01, Fig. 2). There was no difference in intraoperative blood loss (MD = 67.2, 95% CI − 34.8 to 169.1).

#### HHS

**Figure 3:**
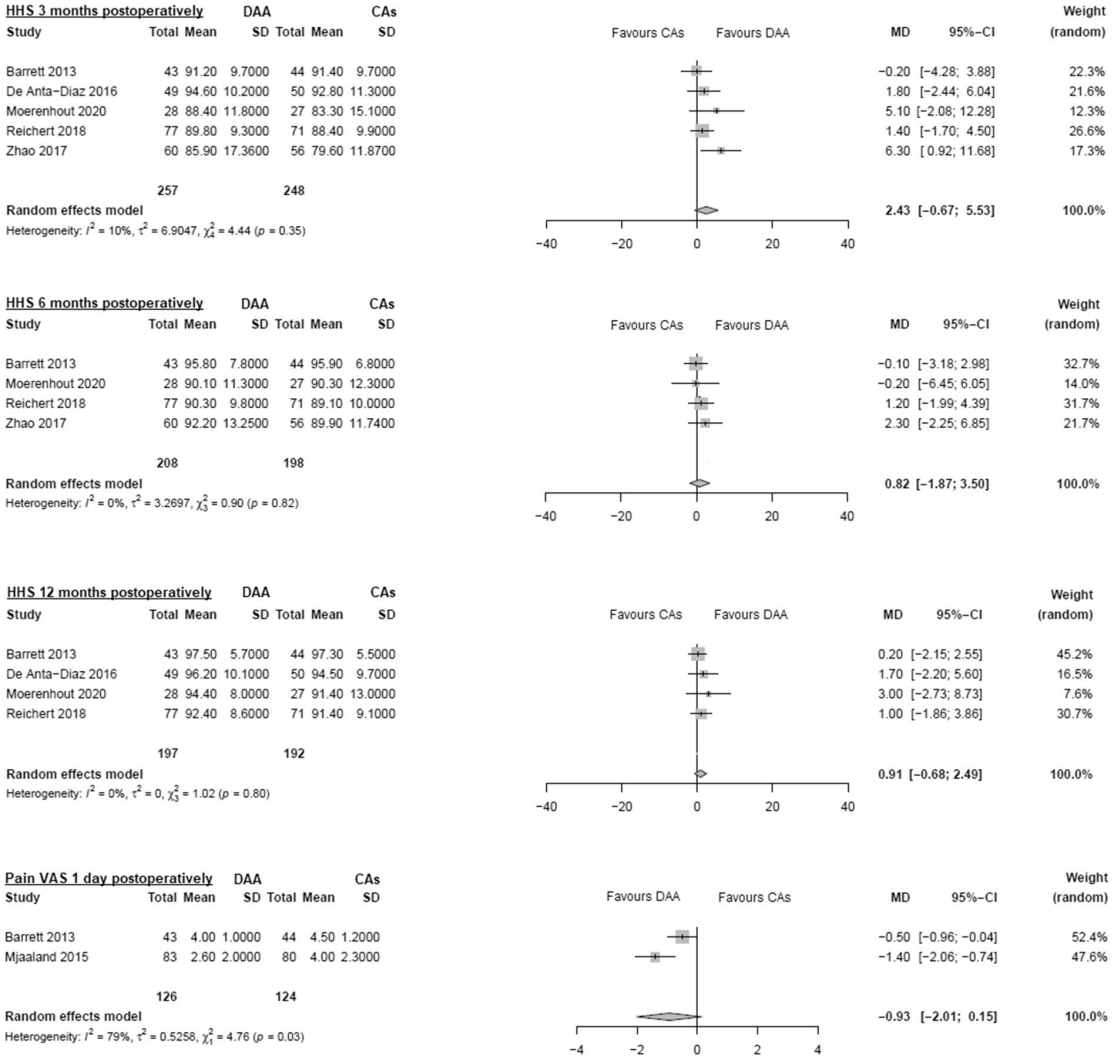
Comparison of the HHS 3, 6 and 12 months postoperatively, pain VAS 1 day postoperatively. *DAA* direct anterior approach, *CAs* conventional approaches, *SD* standard deviation, *MD* mean difference, *CI* confidence interval.

Data on 505 patients were pooled from 5 RCTs (I^2^ = 10%, p = 0.35, Fig. 3). There was no difference in HHS 3 months postoperatively (MD = 2.4, 95% CI − 0.7 to 5.5). Data on 406 patients were pooled from 4 RCTs (I^2^ = 0%, p = 0.82, Fig. 3). There was no difference in HHS 6 months postoperatively (MD = 0.8, 95% CI − 1.9 to 3.5). Data on 389 patients were pooled from 4 RCTs (I^2^ = 0%, p = 0.80, Fig. 3). There was no difference in HHS 12 months postoperatively (MD = 0.9, 95% CI − 0.7 to 2.5).

#### Pain VAS 1 day postoperatively

Data on 250 patients were pooled from 2 RCTs (I^2^ = 79%, p = 0.03, Fig. 3). There was no difference in pain VAS 1 day postoperatively (MD = -0.9, 95% CI − 2.0 to 0.15).

#### Acetabular cup positioning

**Figure 4:**
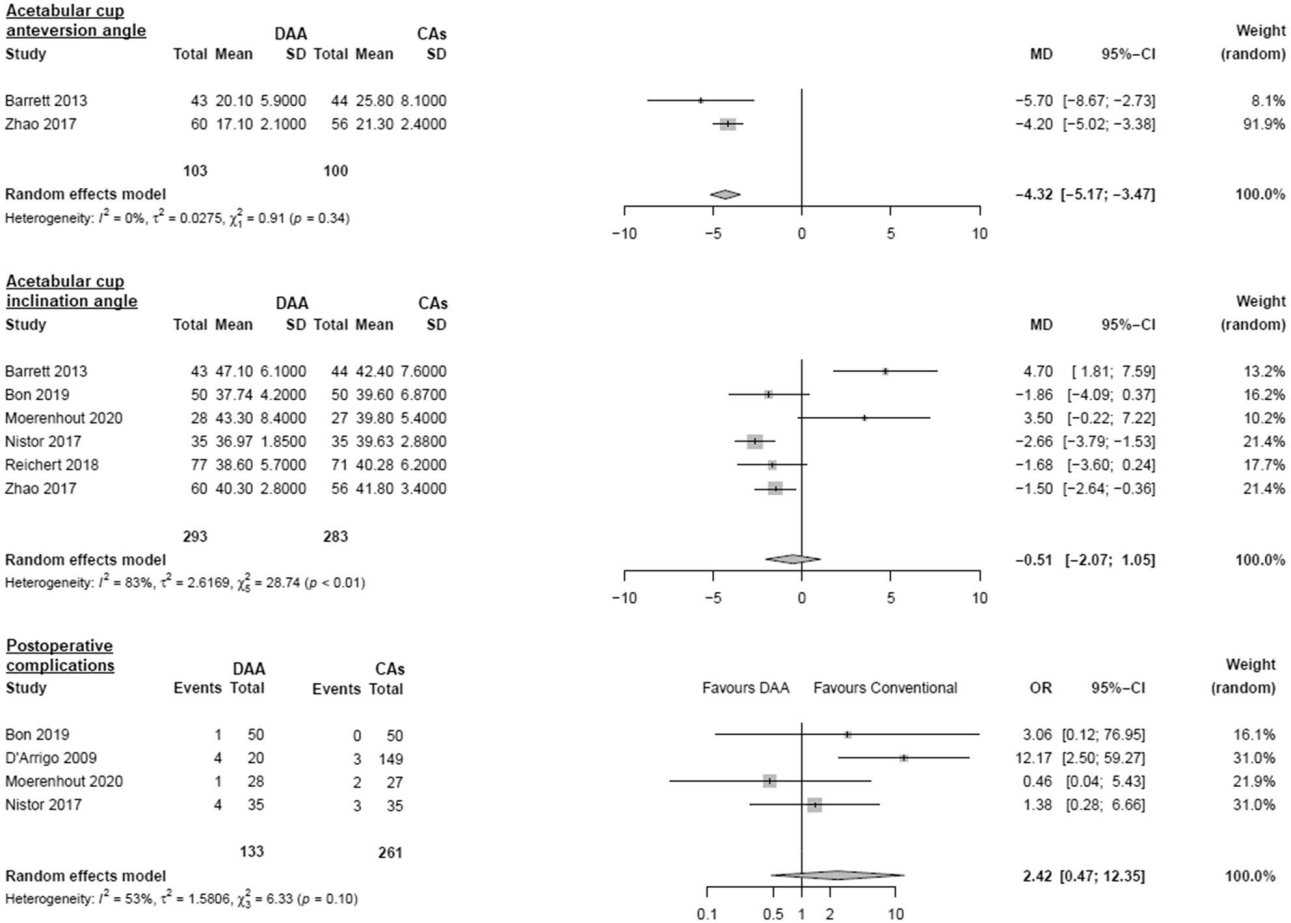
Comparison of the acetabular cup positioning and postoperative complications. *DAA* direct anterior approach, *CAs* conventional approaches, *SD* standard deviation, *MD* mean difference, *CI* confidence interval, *OR* odds ratio.

Data on 203 patients were pooled from 2 RCTs (I^2^ = 0%, p = 0.34, Fig. 4). The acetabular cup anteversion angle of THA through DAA was 4.3° lower than the acetabular cup anteversion angle of THA through CAs (MD = − 4.3, 95% CI − 5.2 to − 3.5). Data on 576 patients were pooled from 6 RCTs (I^2^ = 83%, p < 0.01, Fig. 4). There was no difference in acetabular cup inclination angle (MD = − 0.5, 95% CI − 2.1 to 1.1).

#### Postoperative complications

Data on 394 patients were pooled from 4 RCTs (I^2^ = 53%, p = 0.10, Fig. 4). There was no difference in postoperative complications (OR = 2.4, 95% CI 0.5 to 12.4).

### Traction table subgroup analysis

A traction table was used in three^10,12,25^ out of 10 studies on THA through DAA. The utilization of traction table in THA through DAA showed no influence on the overall effect of the DAA group.

## Discussion

### Main findings

In order to overcome the limitations of the related previous systematic reviews and meta-analyses^1–9^, we conducted our meta-analysis with a more extensive literature search, with restriction to RCTs and with implementation of high-quality statistical methods, testing both fixed and random effects models. Our direct comparison in meta-analysis on short-term outcomes between THA through DAA and CAs included 10 RCTs and 1,053 patients. Four out of 10 studies were blinded RCTs with a level I evidence^10,12,28,31^, the other 6 studies were non-blinded RCTs with a level II evidence^11,25–27,29,30^. THA through DAA showed overall similar surgical, functional, radiological outcomes and postoperative complications compared to THA through CAs. Both approaches showed overall sufficient results in acetabular cup positioning. THA through DAA showed a longer operation time compared to THA through CAs. We found no influence of traction table utilization on outcomes in THA through DAA.

### Operation time

Our meta-analysis found that the operation time of THA through DAA was 17.8 min. longer than the operation time of THA through CAs. The mean operation time varied from 59.9 to 121.0 min. for DAA and from 45.7 to 78.2 min. for CAs. The meta-analyses by Kucukdurmaz et al. ^3^ and Yue et al.^7^ found similarly prolonged operation times in THA through DAA. In contrast, the meta-analyses by Higgins et al.^1^ and Wang et al.^6^ found no difference. Operation time is an outcome parameter that is very difficult to generalize, as there are certainly some confounding factors. During the surgeon's learning curve period, there usually is a prolonged operation time. However, it remains unclear whether THA through DAA were implanted by learners, skilled surgeons, or experts. It is possible to significantly reduce operation time in THA through DAA by acquisition of surgical skills^32^. The wide variation in operation times in THA through DAA from 59.9 to 121 min. supports the idea that the surgeon’s proficiency plays an important role and shows that there is potential for a shorter operation time.

### Incision length

Our meta-analysis found no difference in incision length of THA through DAA compared to THA through CAs. The mean incision length varied from 9.1 to 13.7 cm for DAA and from 10.4 to 14.8 cm for CAs. In contrast, the meta-analyses by Kucukdurmaz et al.^3^ found that THA through DAA had a 3.2 cm shorter incision length than THA through other approaches. The meta-analysis by Wang et al.^6^ showed a 3.5 cm shorter incision length of THA through DAA than THA through posterior approach.

### Intraoperative blood loss

Our meta-analysis found no difference in intraoperative blood loss of THA through DAA compared to THA through CAs. The mean intraoperative blood loss varied from 165.9 to 1344.0 ml for DAA and from 123.8 to 1644.0 ml for CAs. The meta-analysis by Higgins et al.^1^ also showed no difference in intraoperative blood loss. The meta-analysis by Wang et al.^6^ showed a lower postoperative blood loss of 67 ml in THA through DAA compared to THA through posterior approach. The meta-analysis by Yue et al.^7^ showed no difference in postoperative blood transfusion rates between THA through DAA and THA through lateral approach. However, sufficient conclusions about the extent of the tissue trauma cannot be drawn only by determining the intraoperative blood loss. Further outcome parameters such as total blood loss and laboratory parameters such as CRP, IL-6, IL-10, IL-1a, ESR, and CK must also be considered.

#### Harris hip score

Our meta-analysis showed no difference in HHS 3, 6 and 12 months postoperatively in THA through DAA compared to THA through CAs. The mean HHS 3 months postoperatively varied from 85.9 to 94.6 points for DAA and from 79.6 to 92.8 points for CAs. The mean HHS 6 months postoperatively varied from 90.1 to 95.8 points for DAA and from 89.1 to 95.9 points for CAs. The mean HHS 12 months postoperatively varied from 92.4 to 97.5 points for DAA and from 91.4 to 97.3 points for CAs. The meta-analysis by Higgins et al.^1^ examined several different scores (HHS, HOOS, S&R, SF, WOMAC, Oxford Hip Score, Japanese Orthopedic Association Hip Score) describing the functional outcome. The follow-up time for functional outcome varied from 1.5–3 months postoperatively. The overall results showed a weak tendency towards better outcome in THA through DAA compared to THA through posterior approach. The meta-analysis by Kucukdurmaz et al.^3^ found a 5.6 points higher HHS and a 3.1 points lower WOMAC 1.5 months postoperatively in THA through than THA through other approaches. In contrast to the HHS, in WOMAC a lower result is considered as better and a higher result as worse. The meta-analysis by Miller et al.^4^ showed an overall 0.3 point higher HHS in THA through DAA than THA through posterior approach. The HHS was measured at different time points 3 months postoperatively. The network meta-analysis by Putananon et al.^5^ ranked four hip approaches in order from best to fourth best with regard to the HHS 1–1.5 months postoperatively as follows: DAA, lateral approach, posterior and posterior-2-incision approach. THA through DAA had a 2.6 points higher HHS than THA through lateral approach, a 4.8 points higher HHS than THA through posterior approach and a 10.8 higher HHS than THA through posterior-2-incision approach. The meta-analysis by Wang et al.^6^ found that THA through DAA had a 7.4 higher HHS 0.5 months postoperatively than THA through posterior approach and a 6.8 points higher HHS 1.5 months postoperatively than THA through posterior approach. The meta-analysis by Yue et al.^7^ examined several different scores (HHS, SF-36, UCLA, DAQ, WOMAC, LEFS, and LASA) describing the functional outcome. The follow-up time for functional outcome varied from 1.5 months up to years postoperatively. They stated an overall better functional outcome of THA through DAA compared to THA through lateral approach. The improved functional outcome, which several meta-analyses found in THA through DAA, follows the lower tissue and muscle damage, presumably due to operating in a muscle-sparing anatomical plane. Lower tissue and muscle damage in DAA was determined by measuring the levels of relevant laboratory parameters such as CRP, IL-6, IL-10, IL-1a, ESR, and CK in several studies^28,31,33,34^.

### VAS 1 day postoperatively

The mean pain VAS 1 day postoperatively varied from 2.6 to 4.0 points for DAA and from 4.0 to 4.5 points for CAs. Our meta-analysis showed no difference in pain VAS 1 day postoperatively. In contrast, several related meta-analyses found a lower pain VAS in THA through DAA compared to THA through CAs^3,4,6^. The meta-analysis by Kucukdurmaz et al.^3^ found a 1.3 points lower pain VAS 1 day postoperatively in THA through DAA compared to THA through other approaches. The meta-analysis by Miller et al.^4^ found a 0.4 points lower pain VAS in THA through DAA compared to THA through posterior approach, with pain recorded at various time points in a 3 months postoperative follow-up. The meta-analysis by Wang et al.^6^ found a 0.7, 1.5 and 1.6 points lower VAS 1,2 and 3 days postoperatively in THA through DAA compared to THA through posterior approach. The network meta-analysis by Putananon et al.^5^ ranked four hip approaches in order from best to fourth best with regard to the postoperative pain VAS as follows: lateral approach, DAA, posterior and posterior-2-incision approach. The pain was measured at any postoperative time point. The lower pain intensity in THA through DAA probably occurs for reasons similar to the better functional outcome: the lower tissue and muscle damage, presumably due to operating in a muscle-sparing anatomical plane.

### Acetabular cup positioning

The acetabular cup anteversion angle varied from 17.1° to 20.1° in THA through DAA and the acetabular cup inclination angle varied from 37.0° to 47.1° in THA through DAA. The acetabular cup anteversion angle varied from 21.3° to 25.8° in THA through CAs and the acetabular cup inclination angle varied from 39.6° to 42.4° in THA through CAs. Since the ideal values for acetabular cup positioning range from 10° to 25° for anteversion and from 35° to 45° for inclination, both approaches seem to show sufficient results regarding the safe zone with clinically irrelevant differences between each other. However, THA through DAA showed a slight tendency towards a too flat inclination angle (outlier rate: 5%). The related meta-analyses by Higgins et al.^1^, Jia et al.^2^ by Yue et al.^7^ showed no differences in acetabular cup positioning. A 2017 systematic review by Seagrave et al.^33^ found that broad conclusions regarding a definitive target zone for cup positioning in THA still cannot be drawn. The ideal target zone for each patient varies, influenced by several confounding factors. The acetabular cup positioning within a target zone cannot eliminate the risk of dislocation^35^.

### Postoperative complications

Our meta-analysis found no difference in postoperative complications of THA through DAA compared to THA through CAs. Bon et al.^10^ found one case (2%) of dislocation and 8 cases (16%) of neuropraxia of the lateral cutaneous nerve of the thigh in 50 patients, operated through DAA. Complications did not occur (0%) in 50 patients, operated through CAs. Moerenhout et al.^12^ found one case (3.6%) of infection in 28 patients, operated through DAA. Two cases (7.4%) of periprosthetic fracture occurred in 27 patients, operated through CAs. D’Arrigo et al.^26^ found 2 cases (10%) of periprosthetic fracture, one case (5%) of rupture of m. tensor fasciae latae, and 2 cases (10%) of haematoma in 20 patients, operated through DAA. One case (0.7%) of periprosthetic fracture, one case (0.7%) of cup malposition, and one case (0.7%) of infection occurred in 149 patients, operated through CAs. Nistor et al.^29^ found one case (2.9%) of periprosthetic fracture, one case (2.9%) of haematoma and 2 cases (5.7%) of neuropraxia of the lateral cutaneous nerve of the thigh in 35 patients, operated through DAA. Two cases (5.7%) of haematoma, and one case (2.9%) of suture granuloma occured in 35 patients, operated through CAs.

#### Traction table utilization

In subgroup analysis our study showed that there was no influence of traction table utilization on outcomes in THA through DAA. A recent 2020 systematic review on DAA by Sarraj et al. included 44 studies with a total of 26,353 patients^36^. The study found no relevant difference in outcome between traction table versus standard table THA through DAA.

## Limitations

We identified the following limitations to our study: First, the long-term outcomes in THA were not considered. Second, due to insufficient data, important outcome parameters such as hospitalization time, postoperative drainage volume and postoperative gait analysis could not be considered. Third, we did not consider the possible influence of the surgeon operating skills, the utilization of tranexamic acid and anticoagulants, bone cement or the types of implants for THA. Lastly, conventional approaches were summarized in one group, although they differ greatly from one another.

## Conclusion

Considering the overall results of our meta-analysis, we can conclude that THA through DAA showed similar short-term surgical, functional, radiological outcomes and postoperative complications compared to THA through CAs.

## Data Availability

The data are available from the corresponding author upon reasonable request.

## Abbreviations

CI: Confidence intervals
CA: Conventional approach
CK: Creatine kinase
CRP: C-reactive protein
DAA: Direct anterior approach
ESR: Erythrocyte sedimentation rate
HHS: Harris hip score
IL-1a: Interleukin 1a
IL-6: Interleukin 6
IL-10: Interleukin 10
MD: Mean difference
RCT: Randomized controlled trial
RoB: Risk of bias
OR: Odds ratio
THA: Total hip arthroplasty
VAS: Visual analogue scale
